# Umbilical Cord Biomarkers of Nutritional and Metabolic Status in Neonates with Intrauterine Growth Restriction

**DOI:** 10.3390/jcm15031043

**Published:** 2026-01-28

**Authors:** Ioana Hermina Toth, Manuela Marina Pantea, Ileana Enatescu, Angelica Teodora Filimon, Flavia Yasmina Kali, Oana Belei

**Affiliations:** 1 Doctoral School, “Victor Babes” University of Medicine and Pharmacy, 300041 Timișoara, Romania; ioana.toth@umft.ro; 2 Department of Neonatology, Emergency Clinical County Hospital Arad, 310037 Arad, Romania; angifilimon@yahoo.com; 3 Twelfth Department, Neonatology Clinic, “Victor Babes” University of Medicine and Pharmacy, 300041 Timişoara, Romania; 4 Department of Neonatology, “Vasile Goldiș” Western University of Arad, 310048 Arad, Romania; 5 Third Pediatric Clinic, “Louis Turcanu” Emergency Children Hospital, 300011 Timişoara, Romania; flaviastania@yahoo.com; 6 First Pediatric Clinic, Disturbances of Growth and Development on Children Research Center, “Victor Babes” University of Medicine and Pharmacy, 300041 Timişoara, Romania; belei.oana@umft.ro

**Keywords:** IUGR, newborn, neonatal metabolism, micronutrient deficiency, vitamin D, ALP, insulin, C-pep, fetal programming

## Abstract

**Background:** Intrauterine Growth Restriction (IUGR) is associated with a distinct neonatal metabolic profile, attributable to chronic intrauterine nutritional deprivation and suboptimal placental nutrient exchange. Upon delivery, IUGR neonates typically present with depleted nutrient stores, dysregulated endocrine activity, and a spectrum of micronutrient deficiencies, factors that collectively compromise metabolic homeostasis and significantly influence subsequent health trajectories. **Methods:** This narrative review systematically synthesizes the current body of evidence from clinical, biochemical, and translational investigations pertaining to the micronutrient status and pivotal endocrine markers in neonates affected by intrauterine growth restriction. The collected findings were integrated to elucidate metabolic adaptation mechanisms, immediate clinical ramifications, and the potential pathways linking neonatal biochemical patterns to long-term metabolic programming. **Results:** IUGR neonates consistently exhibit reduced cord-blood concentrations of essential micronutrients, including vitamin D, iron (Fe), zinc (Zn), magnesium (Mg), folate (vitamin B9), and cobalamin (vitamin B12), reflecting compromised placental nutrient transfer and limited fetal reserves. Concomitantly, endocrine alterations—most notably reduced insulin (INS) and C-peptide (C-pep) levels—indicate suppressed pancreatic β-cell activity and a prevailing hypoanabolic adaptive state. In parallel, disturbances in mineral metabolism, characterized by lower calcium (Ca) concentrations and increased alkaline phosphatase (ALP) activity, suggest impaired bone mineralization during the critical phase of early postnatal adaptation. Collectively, these biochemical patterns increase vulnerability to early clinical complications such as neonatal hypoglycemia and bone demineralization, disrupt early growth trajectories, and are associated with an elevated long-term risk of insulin resistance and adverse cardiometabolic programming. **Conclusions:** IUGR neonates consistently demonstrate a synergistic interplay of micronutrient deficiencies and adaptive endocrine responses, profoundly impacting immediate postnatal metabolic stability and predisposing them to long-term health challenges. Therefore, early biochemical screening, followed by tailored nutritional and hormonal interventions, may assist restore metabolic balance, promote growth and decrease long term risk for metabolic diseases.

## 1. Introduction

IUGR represents a considerable public health concern, with a global prevalence estimated to range from 5% to 10% of all pregnancies, consequently emerging as a major contributor to perinatal morbidity and mortality. IUGR is defined as the fetus’s inability to achieve its intrinsic genetic growth trajectory. This impediment typically stems from an inadequate supply of essential nutrients, oxygen, and growth-promoting factors, with placental insufficiency being the most common etiological factor. A critical distinction must be drawn between IUGR and the concept of “small for gestational age” (SGA), as not all SGA neonates exhibit pathological conditions; conversely, true IUGR is precisely characterized by concomitant metabolic and hemodynamic compromise.

Increasing evidence has underscored the importance of the Developmental Origins of Health and Disease concept. This paradigm posits that the intrauterine environment exerts a lasting influence on an organism’s structure, function, and metabolic regulation in the postnatal period. IUGR serves as a crucial model within this framework, as fetal adaptations to nutritional and energetic scarcity triggers persistent epigenetic and endocrine alterations. These changes, in turn, predispose individuals to a heightened risk of metabolic disorders, obesity, type 2 diabetes, and cardiovascular disease later in life.

Beyond the established impact of macronutrient deprivation, micronutrient status is increasingly recognized as a pivotal factor in both the pathophysiology and long-term sequelae of intrauterine growth restriction. Deficiencies in essential maternal and fetal vitamins and minerals can impede fundamental biological processes, including cellular proliferation, placental angiogenesis, mitochondrial function, and antioxidant defense mechanisms. Specific micronutrients, such as vitamin D, Fe, Zn, Mg, Ca, vitamins B9 and B12, participate in a multitude of metabolic pathways crucial for orchestrating fetal development, hormonal regulation, and systemic energy homeostasis. Furthermore, the assessment of biochemical markers, including ALP, INS, and C-pep, offers significant diagnostic and prognostic value regarding neonatal metabolic maturation and adaptation to the extrauterine environment.

**Figure 1:**
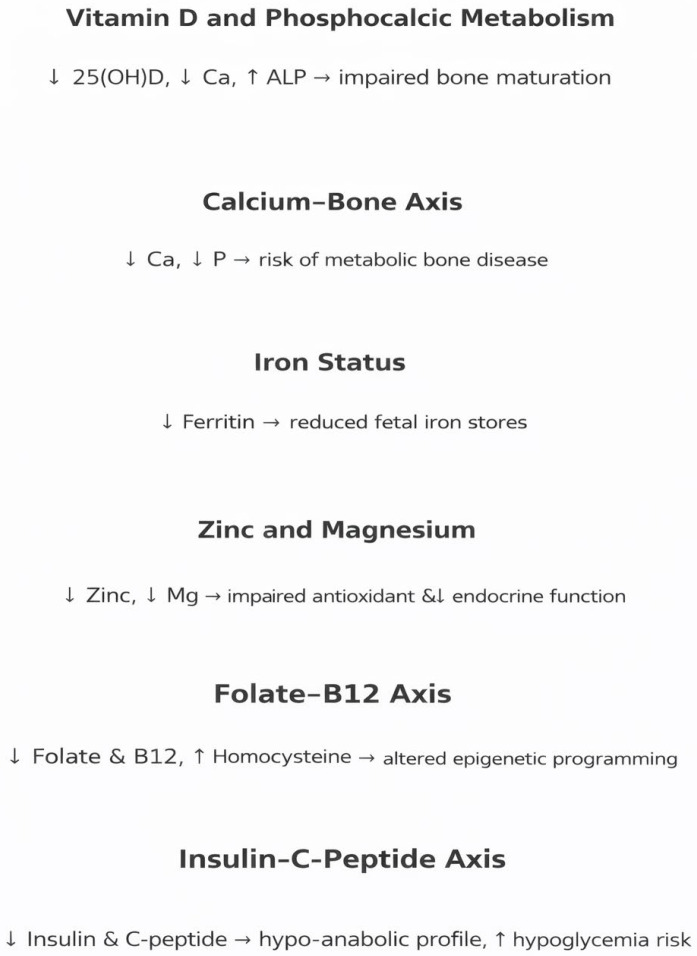
Figure provides an integrated conceptual summary of the biochemical, endocrine, and micronutrient alterations described throughout this review, offering a visual framework for understanding neonatal metabolic adaptation in IUGR (Schematic summary of micronutrient, mineral, and endocrine alterations in IUGR newborns (↑ increase; ↓ decrease).

Contemporary research highlights a frequent association between IUGR and significant changes in micronutrient profiles and metabolic indicators, which directly influence carbohydrate, lipid, and protein metabolism. These metabolic disruptions contribute to the development of neonatal hypoglycemia, modifications in body composition, and an increased susceptibility to accelerated “catch-up” growth in infancy. Such mechanisms are known to foster the premature emergence of insulin resistance and elevate the long-term risk for metabolic complications. The integrated biochemical, endocrine, and micronutrient alterations associated with IUGR are summarized in Figure 1.

The intricate relationship between micronutrient deficiencies and the resultant adaptive metabolic responses in IUGR possesses significant clinical and scientific importance. This review, therefore, endeavors to consolidate current empirical data on the nexus between micronutrient status and metabolic homeostasis in IUGR neonates, elucidate the underlying physiological pathways, and critically evaluate potential nutritional and metabolic interventions for both early prophylaxis and long-term oversight.

Accordingly, we hypothesize that early neonatal profiles of key micronutrients and endocrine markers demonstrate differences between IUGR and AGA newborns. We further hypothesize that there is a link between micronutrient and hormonal alterations to early neonatal complications. These hypotheses contribute to the aim of this review, which aims to synthesize study-level evidence on (i) cord-blood and early neonatal profiles of key micronutrients and endocrine markers in IUGR compared to AGA newborn, (ii) integrating these findings into short- and long-term adverse neonatal biochemical patterns.

Throughout this review, the term vitamin D refers to vitamin D status as assessed by circulating 25-hydroxyvitamin D [25(OH)D] concentrations.

## 2. Materials and Methods

This narrative review was developed using a structured and transparent methodological approach, with the aim of synthesizing current evidence on micronutrient regulation, related metabolic and endocrine biomarkers, and adaptive hormonal and metabolic responses that are altered in neonates with IUGR. The focus is specifically on biochemical measurements in the immediate neonatal period, especially umbilical cord blood, as proxies of intrauterine metabolic conditions and fetal adaptation to placental insufficiency.

### 2.1. Search Strategy

A comprehensive and structured literature search was performed between February 2024 and January 2025 in the following databases: PubMed, Web of Science Core Collection, Scopus, Embase, and the Cochrane Library. The search covered publications from January 2020 to January 2025, reflecting the period in which most advances in neonatal micronutrient biochemistry, perinatal endocrinology, and metabolic programming have been published. Furthermore, limiting included publications from 2020 onwards ensured alignment with recent advances in neonatal clinical nutrition and biomarker technology together with contemporary international recommendations on maternal and neonatal micronutrient management.

The inclusion of the 1998 Cianfarani et al. study is necessary because it establishes the foundational insulin C-peptide in the pathophysiology of IUGR neonates, remaining a reference point for contemporary research. This study demonstrated the combination of low cord C-peptide and insulin-like growth factor-1 (IGF-1) with elevated GH and IGF binding protein-1 in growth-restricted newborns and documenting how this endocrine profile evolves over the first postnatal months. Its inclusion was considered necessary, anchoring for more recent data [1].

Search terms included combinations of MeSH terms and free-text keywords: “intrauterine growth restriction”, “IUGR”, “small for gestational age”, “SGA”, “newborn”, “neonate”, “umbilical cord blood”, “vitamin D”, “Ca”, “ALP”, “Fe”, “ferritin”, “Zn”, “Mg”, “vitamin B12”, “vitamin B9”, “INS”, “C-pep”, “metabolic adaptation”, “endocrine programming”.

Boolean operators (AND/OR) were used to refine the search, and reference lists of eligible articles were screened to identify additional relevant publications.

### 2.2. Eligibility Criteria

Studies were eligible for inclusion if they met the following criteria:

1. Population: Neonates diagnosed with IUGR or SGA, irrespective of gestational age.
2. Outcomes: Reported neonatal biochemical markers—serum or umbilical cord blood concentrations of vitamin D, calcium, ALP, Zn, Mg, Fe, ferritin, vitamin B9, vitamin B12, INS, or C-pep.
3. Design: Prospective or retrospective cohorts, case–control studies, randomized controlled trials, neonatal biochemical surveys, and high-quality systematic reviews
4. Period: Published between 2020 and 2025, except for one historically influential study (1998) included for physiological context.
5. Indexing: Articles published in peer-reviewed journals indexed in Web of Science
6. Language: English.

Exclusion criteria comprised the following: studies reporting only maternal biochemical markers; animal studies; case reports without quantitative laboratory data; conference papers without full peer-reviewed publication; and narrative reviews without primary neonatal data.

### 2.3. Study Selection

All identified records were first assessed at the title and abstract level. Articles meeting initial criteria were retrieved in full text and evaluated against predefined inclusion/exclusion rules. Two independent reviewers screened the studies, and discrepancies were resolved through consensus.

The selection process prioritized neonatal studies providing specific quantitative biomarker data. Special emphasis was placed on studies using umbilical cord blood, as these measurements directly reflect intrauterine metabolic conditions.

### 2.4. Data Extraction and Synthesis

From each eligible publication, the following data were extracted:

- cohort characteristics (sample size, gestational age, sex distribution);
- diagnostic criteria for IUGR/SGA (customized growth charts, population charts, Doppler findings);
- type of biological sample (umbilical cord serum/plasma, neonatal venous blood);
- assay methods (ELISA, chemiluminescence, spectrophotometry, immunoassay);
- mean or median biomarker concentrations, reference ranges, and percentile distributions;
- associations between micronutrient status and outcomes such as birth weight, Apgar score, metabolic complications (hypoglycemia, hypocalcemia), or early postnatal morbidity;
- correlations with maternal factors (nutrition, vitamin supplementation, anemia, gestational diabetes, inflammation).

Due to heterogeneity in study designs, biomarker cut-offs, and population characteristics, a meta-analysis was not performed. Findings were summarized narratively in the following thematic domains:

- vitamin D and phosphocalcic metabolism;
- Ca and ALP physiology;
- Fe and ferritin stores;
- Zn and Mg homeostasis;
- Vitamin B9 and vitamin B12 in one-carbon metabolism;
- INS and C-pep as markers of β-cell activity and metabolic adaptation.

This thematic organization enabled an integrated interpretation of how micronutrient deficiencies and endocrine alterations interact to shape neonatal metabolic outcomes in IUGR.

### 2.5. Quality Assessment

Although no formal scoring tool was applied, each study underwent descriptive quality appraisal. Key aspects considered included the following:

- clarity and consistency of IUGR diagnostic criteria;
- sample size and representativeness;
- use of validated laboratory assays;
- adjustment for confounders such as maternal anemia, diabetes, body mass index (BMI), preterm birth, and inflammation;
- whether cord blood or early neonatal measurements were obtained before significant postnatal metabolic adaptation.

Priority was given to studies with robust methodology, adequate sample sizes, and direct neonatal biochemical measurements.

## 3. Relevant Sections

### 3.1. Vitamin D and Phosphocalcic Metabolism in IUGR

Vitamin D is a key micronutrient in fetal development, and its functions extend well beyond its classical role in calcium and phosphorus regulation. Throughout pregnancy, vitamin D3 supports active transplacental calcium transport and bone mineralization. It also aids in cellular differentiation and modulates immune and endocrine pathways. Growing evidence links maternal vitamin D deficiency with an increased risk of IUGR, preterm birth, and low birth weight [2,3,4].

The placenta plays a fundamental role in vitamin D metabolism. Placental 1α-hydroxylase—an enzyme in the placenta—converts 25-hydroxyvitamin D, the storage form of vitamin D, into the active form, 1,25-dihydroxyvitamin D. This active form acts via the vitamin D receptor (VDR), a protein expressed in trophoblastic and endothelial tissue. In placental insufficiency—a hallmark of IUGR—1α-hydroxylase activity is reduced. VDR expression is also downregulated [2,5]. These changes impair fetal calcium (Ca) and phosphate transport and affect skeletal development and metabolic homeostasis.

Vitamin D also influences energy and glucose metabolism. It modulates insulin secretion and sensitivity, regulates genes involved in protein synthesis and adipocyte differentiation, and affects inflammatory signaling. Maternal vitamin D deficiency correlates with elevated pro-inflammatory cytokines and increased oxidative stress, both contributing to placental dysfunction and restricted fetal growth [6,7].

In growth-restricted neonates, serum 25(OH)D concentrations are frequently low, and disturbances in total and ionized Ca as well as ALP activity are also common [8,9]. These biochemical abnormalities signal delayed bone maturation and impaired phosphocalcic metabolism, and they may predispose to early postnatal hypocalcemia. Transient neonatal hypocalcemia is more common in infants with severe IUGR, underscoring the need for close metabolic monitoring [10].

Recent neonatal studies confirm that IUGR infants exhibit significantly lower 25(OH)D levels than infants who are appropriate for gestational age. Furthermore, there are strong correlations between vitamin D status, birth weight, and the degree of growth restriction [8,10].

Beyond skeletal health, vitamin D plays a role in broader metabolic programming. Low neonatal or maternal vitamin D status has been associated with increased insulin resistance in infancy and early childhood, suggesting that prenatal vitamin D deficiency may contribute to long-term metabolic vulnerability [5,11].

### 3.2. Ca and ALP: Markers of Bone Maturation and Energy Metabolism in IUGR

Calcium is an essential micronutrient for fetal skeletal development, neuromuscular function, and enzymatic activity. Transplacental calcium transfer reaches its peak during the third trimester. When placental insufficiency develops, as in IUGR, this transport becomes impaired. As a result, fetal phosphocalcic homeostasis is disrupted, which contributes to delayed skeletal maturation [12].

Studies on maternal–neonatal dyads and comparisons between IUGR and AGA newborns have revealed distinct patterns of bone turnover. These support the concept of fetal metabolic programming of bone health [12,13]. During the first 48–72 h after birth, IUGR infants—particularly preterm neonates—are at increased risk of transient neonatal hypocalcemia, driven by diminished fetal mineral stores and immature parathyroid responsiveness [12,14]. Current neonatal guidelines recommend assessing ionized Ca, ALP, Mg, albumin, and parathyroid hormone (PTH) to differentiate mineral metabolism disorders [15].

ALP activity is often elevated in the context of impaired bone mineralization—especially when accompanied by hypophosphatemia—a biochemical profile characteristic of metabolic bone disease of prematurity (MBDP). Not all studies identify IUGR as an independent risk factor for MBDP, but this biochemical signature remains clinically valuable for early detection [16,17,18]. Recent cohorts of preterm infants with and without IUGR indicate that bone status is more vulnerable when fetal growth restriction is present. Furthermore, early nutritional strategies, such as Ca/ALP intake, vitamin D supplementation, and milk fortification improve skeletal outcomes [13].

Some paradoxical biochemical presentations—such as hypercalcemia secondary to hypophosphatemia—have been documented in extremely low birth weight small-for-gestational-age infants, underscoring the complexity of mineral regulation during postnatal adaptation and emphasizing the central role of phosphate in growth plate physiology [19].

Overall, serial monitoring of Ca/P/ALP levels and individualized mineral supplementation are essential in IUGR infants to support optimal bone maturation and reduce the risk of long-term complications [13,16].

### 3.3. Fe, Ferritin, and Oxidative Metabolism in IUGR

Iron is one of the essential micronutrients for fetal and neonatal development, with fundamental roles in hemoglobin synthesis, oxygen transport, brain development, and mitochondrial function. During pregnancy, maternal iron requirements progressively increase, particularly in the third trimester, due to expansion of the maternal erythrocyte mass and fetal iron storage. Mother-to-fetus iron transfer occurs via an active process mediated by placental transport proteins, mainly ferroportin (FPN 1), transferrin receptor 1 (TfR1), and hephaestin, all regulated by the systemic iron-regulatory hormone hepcidin.

In placental insufficiency and chronic hypoxia, typical of IUGR, transport mechanisms are markedly disrupted. FPN and TfR1 expression is downregulated, while placental hepcidin levels increase, limiting iron flux to the fetal circulation. Consequently, fetal iron stores—reflected by serum ferritin—are reduced. Large observational studies have demonstrated that both maternal and neonatal ferritin levels are closely associated with birth weight and growth outcomes [20,21,22,23]. In particular, newborns with IUGR display significantly lower cord-blood ferritin levels compared with appropriate-for-gestational-age (AGA) infants, even in the absence of maternal anemia [20,21].

Fetal and neonatal iron deficiency has profound consequences extending beyond compromised oxygen-carrying capacity. Fe serves as a vital cofactor for mitochondrial enzymes integral to oxidative phosphorylation. Its depletion reduces energy production and promotes the accumulation of reactive oxygen species. In the context of intrauterine growth restriction, oxidative stress constitutes a primary mechanism of placental and fetal injury, leading to an imbalance between reactive oxygen species generation and antioxidant defenses. In such cases, labile Fe can exert pro-oxidant effects, catalyzing the Fenton reaction and fostering lipid peroxidation at the cellular membrane level.

Ferritin, in addition to its role as an iron-storage protein, acts as an acute-phase reactant, modulating inflammatory responses. Extremely low ferritin levels in IUGR neonates generally reflect depleted iron reserves, whereas elevated concentrations may indicate systemic inflammation or enhanced oxidative stress. Therefore, accurate interpretation of ferritin in growth-restricted newborns requires concurrent assessment of inflammatory markers and erythropoietic activity [23].

Long-term, fetal Fe deficiency has been associated with cognitive delay, impaired myelination, altered motor development, and increased susceptibility to insulin resistance. These outcomes reflect the broader consequences of fetal metabolic programming.

Moreover, chronic hypoxia and oxidative stress in IUGR stimulate erythropoietin (EPO) production and enhance fetal erythropoiesis, further increasing Fe requirements and intensifying the mismatch between fetal Fe demand and supply [21,22].

Assessment of Fe and ferritin profiles at birth, followed by postnatal hematologic monitoring, is essential for early identification of iron deficiency in IUGR infants.

Appropriately dosed supplementation—adjusted for weight, gestational age, and individual risk factors—can prevent Fe-deficiency anemia and support optimal neurocognitive development. Likewise, maternal nutritional optimization during pregnancy, including adequate dietary Fe and vitamin C intake and reduction in oxidative stress, may contribute to improved fetal iron status and reduced risk of growth restriction.

### 3.4. Zn and Mg: Essential Cofactors in Metabolic Regulation in IUGR Newborns

Zn and Mg act as indispensable cofactors for hundreds of enzymatic reactions involved in protein synthesis, oxidative stress regulation, cellular growth, and energy metabolism. One of the most prevalent trace metals in humans is zinc, which plays a pivotal role in the majority of key metabolic and enzymatic pathways. Zinc interacts with proteins, facilitating enzymatic processes, preserving stability of structures and enhancing binding with other molecules. Additionally, DNA binding proteins depend on zinc to control gene expression. Together with zinc, another vital trace metal is magnesium, which is involved in many metabolic processes such as protein synthesis, mitochondrial mechanisms, neuromuscular activity and immune functionality. Both zinc and magnesium status are essential during the gestation for an optimal development of the fetus and afterwards in the neonatal period. Furthermore, magnesium may regulate disease manifestation in a long-term fashion—in childhood and adulthood [24,25].

At birth, neonatal Zn and Mg levels reflect maternal nutritional status, fetal reserves, and the efficiency of placental transport. In IUGR, clinical evidence consistently demonstrates lower serum or umbilical cord Zn levels compared with AGA newborns, with strong correlations to birth weight and early metabolic vulnerability [26,27,28,29].

Comparative neonatal studies have shown a significant Zn deficit in SGA versus AGA infants—including preterm subgroups—highlighting the need for early metabolic monitoring and potential supplementation [26]. Maternal–fetal paired sampling reinforces these findings; pregnancies complicated by IUGR exhibit a reduced maternal–cord gradient, suggesting impaired placental Zn transfer [27]. Additionally, cord-blood Zn levels have been repeatedly associated with neonatal anthropometric parameters, supporting the clinical relevance of routine Zn assessment at birth [28,29].

Beyond structural and antioxidant roles, Zn directly influences pancreatic β-cell activity and INS secretion. Thus, low Zn levels may contribute to glycemic instability during the transitional metabolic period in IUGR newborns. Zn deficiency has also been linked to impaired immune maturation in preterm infants, amplifying the risk of infection and oxidative damage—mechanisms intimately involved in the pathophysiology of fetal growth restriction [30].

Mg homeostasis in IUGR infants exhibits its own distinct pattern. Studies assessing intracellular Mg—especially in platelets—have demonstrated lower levels in SGA compared with AGA neonates, reflecting diminished intracellular stores and potential effects on neuromuscular excitability and vascular regulation [31]. Additional neonatal research indicates that Mg balance may be altered in the context of placental insufficiency, underscoring its importance in metabolic surveillance [32].

From a clinical standpoint, infants with IUGR or SGA should undergo biochemical evaluation within the first 24–48 h after birth, including serum Zn levels and, when available, magnesium assessment alongside the phosphocalcic profile.

Identifying low zinc concentrations warrants targeted nutritional monitoring and consideration of supplementation, integrated with glycemic and inflammatory biomarkers. Mg interpretation should account for the newborn’s mineral balance and perinatal adaptation, helping clinicians anticipate neuromuscular complications or metabolic instability during early neonatal life.

### 3.5. Vitamin B9 and Vitamin B12: Epigenetic Control and Metabolic Programming in IUGR Newborns

Vitamin B9 and vitamin B12 are essential water-soluble vitamins that play critical roles in fetal growth, DNA synthesis, cellular proliferation, and neurodevelopment. Both function as key cofactors within the one-carbon metabolic pathway, which provides methyl groups necessary for nucleotide formation and for transmethylation reactions. The central product of this pathway, S-adenosylmethionine, serves as the universal methyl donor, modulating gene expression, cellular differentiation, and tissue maturation throughout gestation.

During healthy pregnancy, vitamin B9 and vitamin B12 are actively transported across the placenta via specialized transporters, including folate receptor alpha (FOLR1), reduced folate carrier (RFC), and transcobalamin II (TCN2). Efficient maternal–fetal transfer ensures adequate fetal stores at birth, supporting rapid somatic and neurologic development.

In pregnancies affected by IUGR, placental insufficiency and chronic hypoxemia impair transporter expression and reduce nutrient flux. Studies measuring vitamins B9 and B12 in umbilical cord blood consistently report significantly lower concentrations in IUGR or SGA newborns compared with AGA controls [33,34,35].

This deficiency occurs even when maternal intake is adequate, indicating that transplacental dysfunction—not maternal malnutrition—is often the primary driver.

The reduced availability of Vitamins B9 and B12 compromises nucleotide synthesis, cell division, and tissue expansion, thus contributing directly to impaired fetal growth.

Vitamin B9 and vitamin B12 are required for the remethylation of homocysteine to methionine. In IUGR neonates, impaired one-carbon metabolism is reflected by elevated umbilical cord homocysteine levels, signaling increased oxidative stress and disrupted methylation homeostasis [34].

Deficiencies in these vitamins can induce global and gene-specific hypomethylation, particularly affecting pathways involved in glucose transport, insulin sensitivity, adipogenesis, and lipid metabolism. These alterations may set the stage for long-term metabolic dysfunction, including insulin resistance, obesity, and metabolic syndrome—mechanisms central to the Developmental Origins of Health and Disease (DOHaD) model [35,36].

Furthermore, several neonatal studies have observed associations between low vitamins B9/B12 status and higher cord INS or C-pep concentrations, suggesting a compensatory hyperinsulinemic response to intrauterine nutrient deprivation. This endocrine adaptation underscores the neonate’s attempt to preserve metabolic homeostasis despite restricted growth conditions.

Adequate vitamin B9 and vitamin B12 levels are essential for neurogenesis, myelination, and synaptogenesis. IUGR newborns with low cord concentrations exhibit lower birth weight, reduced head circumference, and lower Apgar scores, and they may face increased risk of cognitive delay and neurodevelopmental disorders during infancy and childhood [33,34].

Given their fundamental metabolic and epigenetic roles, assessing vitamin B9, vitamin B12, and homocysteine levels in umbilical cord blood is recommended for newborns with IUGR or SGA.

Identifying deficiencies should prompt individualized nutritional strategies—promoting breastfeeding and considering targeted supplementation—to optimize metabolic and neurodevelopmental outcomes.

IUGR infants benefit from their mothers’ own milk, as it is a gold standard for nutrition. IUGR, as well as preterm neonates, are high-risk categories that need nutrition-targeted interventions. Human milk composition from IUGR pregnancies is dynamic but not specifically related to IUGR. It is demonstrated that neither macronutrient, nor oligosaccharides or fatty acids, significantly differ from AGA human milk. Current evidence does not support clinically significant or sustained alterations in milk composition from IUGR pregnancies that would compromise infant nutrition during established lactation [37,38,39]. Data for human milk composition related to prematurity emphasizes that the degree of prematurity is the dominant predictor of mother’s milk composition. However, this should not be extended to IUGR cases [40].

At a population level, maintaining optimal maternal vitamins B9 and B12 status reduces the incidence of fetal growth restriction and improves neonatal metabolic profiles, highlighting these micronutrients as modifiable factors in fetal programming [35,36].

### 3.6. The INS–C-Pep Axis in IUGR Newborns

INS plays a central role in fetal growth and energy regulation, functioning both as a key metabolic hormone and a potent anabolic growth factor. During intrauterine life, INS stimulates glucose uptake, amino acid utilization, lipogenesis, and protein synthesis, directly contributing to somatic growth and organ maturation. C-pep, released in equimolar amounts with INS, serves as a stable and specific marker of endogenous β-cell activity, offering a reliable indicator of fetal pancreatic function independent of INS clearance.

In normal pregnancies, INS concentrations rise progressively with gestational age, reflecting maturation of fetal β-cells and adequate transplacental nutrient delivery.

In contrast, placental insufficiency—a hallmark of IUGR—limits fetal nutrient and oxygen supply, prompting an adaptive endocrine response. This adaptation includes suppressed INS synthesis and reduced β-cell responsiveness, reflecting a strategy to conserve energy and maintain euglycemia under conditions of restricted substrate availability [1].

Comparative studies between infants with IUGR or SGA status and appropriate-for-gestational-age AGA controls consistently show significantly lower levels of INS and C-pep in umbilical cord blood [41,42,43].

These reduced concentrations reflect a diminished β-cell secretory capacity and correlate positively with birth-weight percentile, placental weight, and lean body mass—underscoring INS’s critical role in fetal growth.

Conversely, in pregnancies complicated by maternal hyperglycemia or gestational diabetes, the opposite metabolic profile is observed—elevated cord INS and C-pep levels, indicative of fetal hyperinsulinism and increased anabolic drive. These contrasting patterns highlight the dual and context-dependent function of INS in intrauterine growth.

Recent evidence strengthens this conceptual model. A 2025 Diabetes Care cohort demonstrated that cord C-pep concentrations vary systematically according to maternal metabolic phenotype, with the lowest values observed in INS-sensitive pregnancies—which often overlap with fetal growth restriction—and the highest levels seen in INS-resistant gestational diabetes [44].

Complementary findings presented at the 2025 ADA Scientific Sessions showed that fetal polygenic risk scores for β-cell function correlate with both C-pep levels and birth-weight percentile, suggesting that genetic determinants of insulin secretion capacity are active even before birth [45].

A 2025 Diagnostics review emphasized that cord-blood C-pep represents a robust independent biomarker of fetal metabolic adaptation, useful in distinguishing hypoinsulinemic IUGR profiles from hyperinsulinemic states such as diabetic pregnancies [46].

Low INS and C-pep levels in IUGR newborns reflect a hypo-anabolic metabolic state, clinically manifested by reduced glycogen reserves, diminished adipose tissue, lower birth weight, and increased susceptibility to neonatal hypoglycemia.

Although this endocrine suppression is adaptive during fetal life, it may predispose to long-term alterations in metabolic programming. Longitudinal studies reveal higher risks of insulin resistance, altered glucose homeostasis, and cardiometabolic disease in individuals born with IUGR—findings aligned with the Developmental Origins of Health and Disease (DOHaD) framework [41,42,43,44,45,46].

Micronutrient deficiencies frequently present in IUGR—particularly Zn, MG, Fe, vitamin D3, vitamin B9, and vitamin B12—may further exacerbate impaired β-cell function by disrupting INS synthesis, mitochondrial efficiency, and receptor signaling. Conversely, adequate maternal micronutrient intake and improved placental perfusion are critical for supporting fetal endocrine development.

In the neonatal period, measurement of cord-blood INS and C-pep, alongside glucose and micronutrient panels, provides essential insight into the metabolic adaptation of IUGR infants.

Low C-pep values should prompt intensified glycemic monitoring and individualized nutritional management to prevent hypoglycemia and excessive catabolic stress.

Integrating endocrine findings with maternal and neonatal micronutrient status helps differentiate between primary β-cell immaturity and secondary functional suppression due to nutrient deprivation.

Preventive maternal strategies—including adequate intake of vitamin D3, Fe, vitamin B9, vitamin B12, Zn, and Mg—support placental function and facilitate optimal fetal endocrine maturation [42,44,45,46].

An overview of the cord-blood and early neonatal biochemical and endocrine differences between AGA and IUGR/SGA newborns, synthesizing the main micronutrient, mineral, and hormonal alterations discussed in Section 4.1, Section 4.2, Section 4.3, Section 4.4, Section 4.5 and Section 4.6, is presented in Table 1.

### 3.7. Summary

IUGR newborns demonstrate a distinct INS–C-pep profile marked by reduced β-cell secretory activity and diminished anabolic signaling. These hormonal characteristics reflect adaptive responses to chronic intrauterine undernutrition but simultaneously establish a metabolic imprint predisposing to later INS resistance, glucose intolerance, and cardiometabolic vulnerabilities.

Cord-blood INS and C-pep measurements, combined with micronutrient assessment, form a cornerstone of early metabolic characterization in growth-restricted infants and provide a framework for targeted postnatal interventions aimed at supporting endocrine recovery and promoting healthy growth trajectories.

## 4. Discussion

IUGR represents a significant challenge within neonatal medicine, reflecting the cumulative effects of chronic fetal undernutrition, placental insufficiency, and complex adaptive endocrine–metabolic responses. By contrast, the broader SGA population also includes constitutionally small infants, yet remains metabolically and hemodynamically normal. Distinguishing IUGR from SGA is important, as only SGA infants with abnormal Doppler findings, placental disease, or metabolic compromise meet criteria for true IUGR. This matters because pathophysiological IUGR is characterized by a coordinated fetal adaptive response—brain sparing redistribution of blood flow, reduced liver perfusion, and selective restriction of muscle and subcutaneous tissue growth—that is not observed in healthy SGA newborns [48].

Upon birth, IUGR neonates exhibit a distinctive physiological and biochemical phenotype, characterized by diminished adipose and glycogen reserves, reduced lean muscle mass, and dysregulated endocrine and metabolic functions. This characteristic pattern arises from chronic intrauterine undernourishment, hypoxemia and the redistribution of the blood flow ensuing downregulation of the anabolic liver and muscle pathways. The aforementioned features underscore the metabolic compromises initiated during intrauterine life. While such adaptations initially enhance fetal survival in a nutritionally restricted environment, they frequently become maladaptive postnatally, predisposing the infant to metabolic instability, transient hypoglycemia, and impaired growth trajectories [49].

This discussion focuses on the micronutrient–endocrine–metabolic axes, the network of interactions that characterizes the IUGR neonatal population, rather than the SGA neonates. It examines how cord-blood deficiencies in vitamin D, zinc, magnesium, iron, vitamin B9, and vitamin B12 are related to placental failure or maternal intake. Combining deficiencies with perturbations in the INS–C-pep and Ca–phosphate axes, they collectively contribute to neonatal homeostatic alterations and to a higher long-term risk of metabolic diseases.

### 4.1. Micronutrient Deficiency and Neonatal Nutritional Vulnerability

Neonates affected by intrauterine growth restriction frequently present with depleted nutritional stores and exhibit biochemical indicators of micronutrient deficiencies, regardless of the adequacy of maternal nutritional status. This is largely attributable to inefficient placental nutrient transfer, which leads to reduced cord-blood levels of essential micronutrients such as vitamin D, Zn, magnesium, and Fe, thereby directly influencing neonatal metabolic processes and organ development.

Micronutrient deficiency in IUGR neonates shapes neonatal nutritional vulnerability relying on some chore mechanisms, such as placental transport failure, oxidative stress, mitochondrial dysfunction, endocrine pathway and epigenetics. First to be described here is placental insufficiency, also referred as impaired delivery of nutrients and oxygen. Fetal hypoxia is related to the reduced placental blood flow, which is primarily linked to placental oxygen transfer. As for transplacental transport of nutrients, this is rather linked to transporter proteins expressed in the syncytiotrophoblast plasma membranes. Based on the regulation by maternal supply model, the placenta responds to maternal signals such as undernutrition, stress, and hypoxia, therefore causing alterations in allocating resources to the fetus, such as vitamin D, Zn, magnesium, and Fe. Nevertheless, there are also complex signaling pathways in the placenta—insulin/IGF, mTOR—that are involved in maternal physiology and fetal development. These keys signaling pathways are linked to placental insufficiency and may lead to low fetal micronutrient storage even with adequate maternal nutrition intake. Regardless of the causal mechanism, low micronutrient levels directly limit the substrate for brain, bone and endocrine development [50]. Oxidative stress and mitochondrial dysfunction are consistently associated with IUGR. Oxidative stress results from redox imbalance defined by high reactive oxygen species (ROS) or low antioxidant activities. Mitochondrial changes at the placental level include critical dysfunction on energy metabolism, oxidative balance and cell survival. Both mechanisms are involved in long-term adverse effects in IUGR neonates, suggesting insulin resistance and cardiac remodeling. Low levels of iron and zinc may contribute to impaired mitochondrial functions increasing products of oxidative stress [51,52,53].

Regarding endocrine mechanisms, the glucose–insulin pathway is one key factor associated with metabolic sequelae in IUGR population. In IUGR, pancreatic islets face a dysfunction marked by capillary rarefaction, driven by oxidative stress, which disrupts vascular endothelial growth factor (VEGF) and nitric-oxygen-dependent endothelial–β-cell crosstalk, reducing β-cell proliferation and leading over time to loss of β-cell mass. This dysfunction alone cannot be pointed to as responsible for impairments associated with IUGR. Insulin secretion also plays a role. β-cell metabolism of glucose generates reactive oxygen species, especially H_2_O_2_ from mitochondria and NADPH oxidase, which acts as a physiological messenger in glucose-stimulated insulin secretion. However, reducing reactive oxygen species with antioxidants impairs insulin secretion. Naturally, β-cells show low antioxidant enzyme expression, which favor redox signaling and leaves them highly vulnerable to oxidative damage. Nevertheless, pancreatic insulin secretion is only one determinant of glucose homeostasis, since insulin-mediated glucose uptake in peripheral tissues is the main mechanism for maintaining glycemia [53]. The two main insulin-responsive peripheral tissues are the liver and the muscle. Hepatic and skeletal muscle insulin resistance develops before overt hyperglycemia, with impaired insulin signaling through the insulin receptor–PI3K–AKT pathway reducing glycogen synthesis in liver and GLUT4-mediated glucose uptake in muscle. Interestingly, the development of insulin resistance in IUGR is independent of the weight, more related to enzymatic pathways [53,54].

Intrauterine disorder in IUGR leads to islet mitochondrial dysfunction, oxidative stress, and reduced β-cell function staring in utero and persisting lifelong, predisposing patients to metabolic diseases such as type 2 diabetes. Notably, there are some micronutrients and vitamins that may interfere with the glucose-insulin pathways. Magnesium, one important enzymatic cofactor for carbohydrate metabolism, plays a role in insulin resistance. Magnesium deficiency is linked to impaired glucose tolerance and diminished insulin secretion [25,31].

Vitamin D plays a pivotal role during pregnancy, supporting not only placental function, but also reduces inflammation and improves mitochondrial function, leading to better neonatal outcomes. Deficiency of vitamin D is closely linked to impaired placental functioning mirroring adverse outcomes such as IUGR. During pregnancy, vitamin D deficiency contributes to decreasing vascular endothelial growth factor expression in the placenta, which may lead to insufficient vascular development and eventually to IUGR and preeclampsia. Additionally, vitamin D is essential in regulating trophoblast invasion, which is inhibited by low levels of vitamin D. Vitamin D deficiency reduces placental 11β-HSD2 expression, diminishing the placenta’s ability to inactivate maternal glucocorticoids and thereby increasing fetal exposure to stress hormones. This heightened glucocorticoid exposure, often accompanied by elevated maternal glucocorticoid levels, can disrupt normal fetal growth and development and may promote placental inflammation and vascular insufficiency. Furthermore, Vitamin D deficiency promotes a pro-inflammatory placental environment, with increased expression of cytokines (TNF-α, IL-17α, IFN-γ) and chemokines (MCP-1, MIP-2, KC). These adverse structural and inflammatory changes are partially reversible, as supplementation with active vitamin D restores placental growth, improves nutrient transporter expression, and attenuates inflammatory marker expression. Vitamin D is also implicated in maintaining physiological levels of calcium and phosphorus linked to neuromuscular signaling and bone mineralization. Deficiency of vitamin D disrupts calcium transport, vitamin D receptor signaling and enzymatic pathways, compromising both bone mineralization and insulin secretion [55,56].

Maternal nutrition plays a significant role in regulating placental development and optimal fetal growth and development, influencing core mechanisms such as the one-carbon cycle and epigenetics. Micronutrients like folate, vitamin B12 and long-chain polyunsaturated fatty acids are linked through the one-carbon cycle, which regulates gene methylation important for placental and fetal development. The primary form of dietary folates is 5-methyltetrahydrofolate, an important factor in the one-carbon cycle. It donates a methyl group to homocysteine with vitamin B12 as a cofactor to form methionine. Methionine generates S-Adenosyl methionine, which is the universal methyl donor for DNA, RNA, proteins, phospholipids, and hormones. Further, it is converted to s-adenosylhomocysteine and to homocysteine. Homocysteine enters the transsulfuration pathway with vitamin B6 as a cofactor and converts into cysteine. These pathways are marked by folate and vitamin B12 as methyl donors for nucleic acids, neurotransmitters and phospholipids. Through these roles, folate and vitamin B12 support normal placental development, hence fetal development. Imbalanced maternal nutrition, with altered levels of one-carbon metabolites and long-chain polyunsaturated fatty acids, lead to alteration in the one-carbon cycle, thus epigenetic modifications in the placenta leading to adverse pregnancy outcomes such as preeclampsia and IUGR, as well as increased risk of chronic diseases later in life [57,58].

- Vitamin D deficiency impairs Ca absorption and endocrine function, thereby compromising both bone mineralization and pancreatic maturation.
- Fe and ferritin depletion restrict hemoglobin synthesis and mitochondrial activity, which contributes to anemia and heightened oxidative stress.
- Deficiencies in Zn and Mg lead to impaired enzymatic pathways and disruptions in glucose metabolism.
- Inadequate levels of vitamin B9 and vitamin B12 alter one-carbon metabolism,
- subsequently affecting DNA synthesis and methylation processes.

These collective alterations compromise the neonate’s physiological capacity to maintain energetic homeostasis and efficiently navigate the metabolic transition from intrauterine to extrauterine life.

### 4.2. Endocrine Adaptation: The INS–C-Pep Axis in Neonatal Energy Homeostasis

In neonates affected by IUGR, the INS–C-pep axis plays a crucial role in early metabolic adjustment and a sensitive marker of pathophysiological pathway. Investigations of cord blood consistently reveal diminished INS and C-pep concentrations in comparison to AGA peers, reflecting attenuated pancreatic β-cell function, impaired glucose-stimulated insulin secretion, and restricted anabolic signaling. Alterations in the insulin-like growth factor axis constitute a potential marker for IUGR. Low levels of insulin-like growth factor in cord blood in IUGR neonates indicates a reflection of the placental environment [59]. Limes et al. developed an experimental model showing that placental insufficiency and low oxygen levels are linked to elevation of fetal catecholamines, thus inhibiting β-cells impairing fetal insulin secretion [60].

This suppression of insulin secretion is an adaptive fetal strategy that preserves glucose for vital organs and basal metabolic rate under conditions of restricted supply. Notwithstanding, after birth the same endocrine profile remains maladaptive with low insulin and C peptide in combination with reduced hepatic glycogen stores and limited adipose tissue, predisposing IUGR neonates to hypoinsulinemic hypoglycemia, and impaired peripheral glucose uptake [54,61].

Recent data confirm that cord-blood C-peptide levels integrate both genetic β-cell function and the maternal metabolic milieu, with higher values in pregnancies complicated by obesity or gestational diabetes and the lowest values in insulin-sensitive, growth-restricted pregnancies. Cord C-peptide is also associated with newborn adiposity and later childhood fat mass, emphasizing its utility as an early biomarker of endocrine programming and future metabolic risk [46,62].

In IUGR infants, persistent alterations in insulin secretion (via epigenetic repression of key β-cell transcription factors such as PDX-1) and changes in insulin receptor and post-receptor signaling in liver, muscle, and adipose tissue may together promote disproportionate catch-up fat accretion and progressive insulin resistance, even when early glucose levels normalize. These mechanisms help explain why individuals born growth-restricted exhibit a characteristic trajectory from neonatal hypoinsulinism to childhood and adult insulin resistance, central adiposity, and elevated cardiometabolic risk, making early characterization of the INS–C-pep axis in IUGR neonates clinically and prognostically important [62,63,64,65].

### 4.3. Micronutrient–Hormonal Interactions in the Neonate

The interplay between micronutrient status and endocrine function is profoundly intertwined in neonates affected by intrauterine growth restriction.

Zn and Mg are structural and functional cofactors for insulin synthesis, storage, and receptor signaling; Zn is concentrated in β-cell secretory granules, where the ZnT8 transporter imports zinc to form insulin–zinc hexamers essential for proper insulin processing, crystallization, and regulated secretion, while Mg serves as a cofactor for ATP-dependent kinases that mediate insulin receptor autophosphorylation and downstream PI3K–Akt signaling in liver and muscle. Zn and Mg further shape the IUGR endocrine–metabolic phenotype through their roles in antioxidant defense and insulin action. Low Zn reduces metallothionein and Cu/Zn-SOD activity and disrupts ZnT8-dependent insulin granule maturation, while Mg deficiency impairs insulin-stimulated TRPM6 channel activity and worsens insulin resistance, findings supported by improved glycemic indices and oxidative stress markers in pregnant women receiving Mg supplementation. Deficiencies in these elements exacerbate β-cell stress, limit insulin secretory plasticity during catch-up growth, and promote inefficient glucose utilization, thereby amplifying the risk of early hypoglycemia and later dysglycemia in IUGR survivors [31,53,66,67,68].

Maternal and neonatal vitamin D deficiency thus constitutes a major risk factor for IUGR and its associated metabolic sequelae. Beyond correlating with fetal growth restriction, low vitamin D status impairs placental vascularization and nutrient transport, attenuates skeletal mineralization, and reduces β-cell functional reserve, thereby weakening the neonate’s ability to handle postnatal glycemic and mineral stress. Routine assessment of vitamin D status in high-risk pregnancies, coupled with carefully titrated supplementation, is therefore a key strategy to prevent both metabolic bone disease and early endocrine vulnerability in IUGR newborns [11,55,56,69].

Fe and ferritin play a dual role in intrauterine growth restriction, serving as indicators of fetal iron reserves and active participants in oxidative and metabolic adaptations; disrupted fetal iron homeostasis in IUGR is associated with increased placental and neonatal oxidative damage, impaired myelination, altered mitochondrial function, and higher risk of later neurocognitive and metabolic disorders, justifying comprehensive hematologic monitoring and timely iron correction [21,52,53,69].

Collectively, these micronutrient–endocrine interactions mean that the endocrine phenotype of IUGR neonates is a coordinated yet fragile construct, in which small perturbations in vitamin D, Fe, Zn, Mg, B9, or B12 can tip the balance from adaptive survival toward metabolic decompensation, underscoring the importance of early micronutrient profiling and individualized supplementation strategies in this high-risk group.

### 4.4. Bone Mineral Metabolism and Phosphocalcic Adaptation

The Ca–phosphate axis is another critical facet of neonatal adaptation, particularly in infants with intrauterine growth restriction. In the healthy fetus, up to 80% of Ca and phosphate accretion occurs during the third trimester, driven by active placental transport; in IUGR—especially when preterm—this process is curtailed by placental insufficiency and early delivery, resulting in significantly reduced mineral stores at birth. As a consequence, IUGR neonates, particularly those born prematurely, face an elevated risk of transient neonatal hypocalcemia and metabolic bone fragility, arising from both diminished intrauterine mineral accretion and an immature parathyroid hormone (PTH) response that cannot immediately compensate for the abrupt cessation of placental Ca supply [13,18,70,71].

Biochemically, these infants often exhibit low or low-normal serum phosphate and ionized Ca in conjunction with secondary hyperparathyroidism, which promotes skeletal Ca mobilization at the cost of bone mineral density. Elevated ALP levels are frequently observed, reflecting compensatory osteoblastic activity and serving as early indicators of bone mineralization deficits. Implementing early nutritional interventions, specifically ensuring adequate intake of Ca, phosphate, and vitamin D, demonstrably improves skeletal outcomes and reduces the long-term risk of osteopenia or other metabolic bone diseases [13,72,73].

Implementing early nutritional interventions, specifically ensuring adequate intake of Ca, phosphate, and vitamin D, demonstrably improves skeletal outcomes and reduces the long-term risk of osteopenia or other metabolic bone diseases [13].

These observations underscore that mineral metabolism perturbations in IUGR are not merely discrete phenomena but rather an integral component of the extensive metabolic adaptive processes characteristic of neonates affected by IUGR.

### 4.5. Epigenetic Programming and Long-Term Metabolic Impact

Epigenetic programming in IUGR neonates reflects stable molecular changes—influenced by micronutrient status and endocrine milieu—that reset pathways for glucose, lipid, and vascular regulation long before birth. Adverse intrauterine conditions such as placental insufficiency and micronutrient deficits alter DNA methylation and histone markers in key metabolic genes, modifying their expression without changing the DNA sequence. Epigenome studies in cord blood from IUGR neonates have identified impairment in genes involved in growth, insulin signaling, mitochondrial function, and vascular biology, hence leading to metabolic disease later in life [74,75].

Within the Developmental Origins of Health and Disease framework, these epigenetic modifications provide explanations for the relationship between IUGR and higher lifetime risk of insulin resistance, obesity, hypertension, and cardiovascular disease, independent of postnatal weight alone. Therefore, an IUGR neonate should be recognized beyond restricted growth, but rather a reprogrammed mechanism warranting long-term cardiometabolic surveillance and targeted preventive strategies [74,75,76].

### 4.6. Postnatal Nutritional Interventions

Pointing attention to the physiological rationale for tailored and intensified postnatal nutrition in IUGR is strong. These categories of neonates show reduced fat-free mass, depleted mineral and micronutrient stores, and a hypoanabolic endocrine profile. Higher early intakes of energy, protein, vitamin D, iron, calcium and other micronutrients may be necessary for supporting growth and development. Fat-free mass at term-equivalent age correlates better with brain volumes and neurodevelopment than simple anthropometry, supporting a strategy that prioritizes lean-mass accretion rather than weight alone and justifying higher early protein-energy intake [77,78]. However, rapid catch-up growth in IUGR infants is linked to later obesity, insulin resistance and metabolic syndrome, suggesting that both under- and over-nutrition postnatally may be harmful [49,77]. Strong recommendations on high protein-energy intake derive from preterm, specifically data for IUGR is limited. Observational data that highlight the need for tailored nutrition come from IUGR preterm neonates [79].

Enteral nutrition should include the mother’s own milk, if possible, although tailored approaches may be needed. Individualized milk fortification may meet nutrient needs, improving macronutrient intake, and weight gain. ESPGHAN’s recommendation on nutritional management and growth assessment for infants born IUGR is the same as for those born AGA [80]. Parenteral nutrition is another key element that is rather studied specifically in IUGR preterm than IUGR subgroup. Longer parenteral nutrition and delayed enteral feeding correlate with complications like necrotizing enterocolitis, cholestasis and poorer bone health [13,81,82].

Postnatal nutritional management targeted at IUGR infants faces gaps. Many studies use SGA as a synonym for IUGR, which brings misclassification. Most interventions come from preterm or low-birth-weight neonates without IUGR-specific reporting. Randomized control trials directly testing different postnatal regimens in IUGR infants with long-term neurocognitive and cardiometabolic outcomes are lacking, although outcomes like body-composition, bone status, and neurodevelopmental trade-off in IUGR population are available in cohorts [13,77,78].

### 4.7. Clinical and Preventive Implications for the Neonatal Period

The elucidation of the neonatal metabolic profile in IUGR carries significant clinical implications.

- Early evaluation of growth-restricted infants should incorporate comprehensive biochemical screening.
- Targeted nutritional interventions, encompassing vitamin D and mineral supplementation alongside fortified feeding, demonstrably support postnatal growth and promote optimal bone development.
- Sustained glycemic and metabolic surveillance is crucial for the timely identification of hypoinsulinemia or dysglycemia, thereby enabling the implementation of individualized therapeutic strategies.
- Longitudinal assessment of growth trajectories and metabolic parameters is imperative for the early detection of insulin resistance or alterations in body composition, facilitating the provision of personalized care.

The implementation of such proactive management strategies reframes the clinical approach to IUGR infants, transitioning from reactive symptom control to a model of preventive metabolic intervention, with the goal of optimizing both immediate postnatal adaptation and long-term health outcomes.

### 4.8. Summary

**Figure 2:**
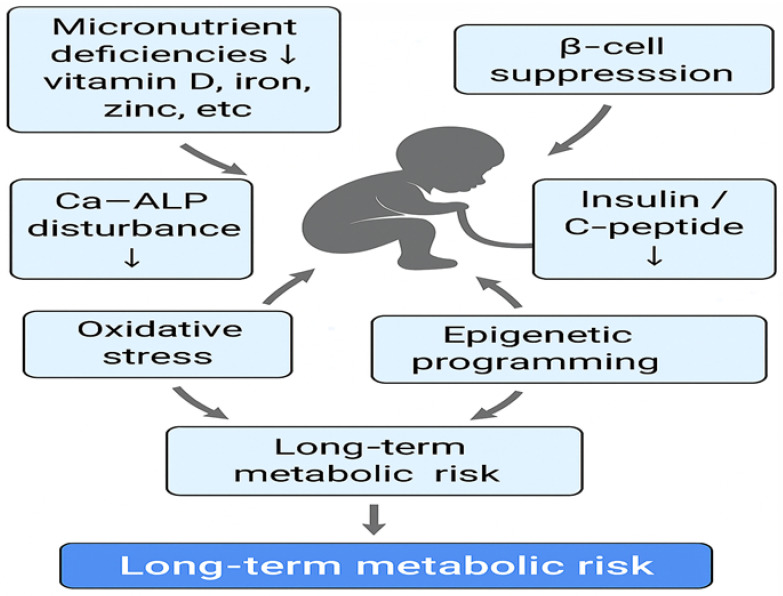
This schematic illustration summarizes the interconnected biochemical and endocrine alterations characterizing IUGR neonates. IUGR is associated with micronutrient deficiencies, altered Ca–ALP homeostasis, oxidative stress, β-cell suppression, and reduced INS and C-peptide levels, contributing to epigenetic programming and increased long-term metabolic risk. The downward arrows indicate decreased levels or impaired activity of the corresponding parameters, namely Ca–ALP homeostasis and INS/C-peptide secretion.

Neonates affected by IUGR present with a distinct metabolic profile, primarily influenced by prenatal nutritional insufficiency and compromised placental function. The proposed mechanisms linking IUGR to long-term metabolic risk are summarized in Figure 2.

Their capacity for postnatal physiological adjustment is critically contingent upon an intricate equilibrium among micronutrient status, endocrine regulation, and adaptive metabolic reprogramming. Prompt identification of this complex interaction facilitates the implementation of expeditious nutritional and endocrine therapeutic strategies, thereby re-establishing physiological equilibrium, promoting optimal growth trajectories, and mitigating the long-term predisposition to chronic metabolic disorders.

## 5. Discussion and Perspectives

### 5.1. Evidence Gaps in the Literature

Facing a growing interest in cord-blood biomarkers and micronutrient panels in IUGR neonates, several gaps remain in the primary literature. Most studies use single cord-blood measurements rather than serial sampling, limiting insights into the trajectories and dynamics of these biomarkers and micronutrients. Furthermore, randomized controlled trials and longitudinal studies related to IUGR population are scarce in the current literature. Most available data derive from other neonatal categories, such as SGA or preterm infants with IUGR, supporting extrapolation for interventional nutritional strategies. Moreover, there is considerable heterogeneity in IUGR definition and SGA overlap, assay methods, and reference ranges for neonatal micronutrients and hormones, hampering cross-study comparability and the derivation of IUGR-specific cut-offs.

Addressing these gaps requires IUGR-focused prospective cohorts and randomized trials, to enable a more precise understanding of this neonatal population.

### 5.2. Conclusions and Future Directions 

IUGR neonates constitute a unique clinical and metabolic cohort within neonatal medicine. Their physiological profile is shaped by the compounded effects of chronic intrauterine undernutrition, placental insufficiency, and adaptive endocrine suppression, culminating in neonates characterized by diminished nutrient reserves, dysregulated hormonal signaling, and heightened metabolic vulnerability.

At birth, these neonates encounter distinct physiological hurdles, including diminished glycogen and lipid stores, impaired insulin secretory capacity, prevalent micronutrient deficiencies, and disruptions in bone mineralization and Ca-phosphate equilibrium.

Collectively, these elements critically undermine their capacity to sustain energy homeostasis and facilitate appropriate postnatal development.

The documented aberrations in concentrations of vitamin D, Zn, Fe, Mg, vitamin B9, and vitamin B12, alongside reduced INS and C-pep levels, signify a compensatory endocrine-nutritional adaptation that, while critical for fetal viability, ultimately elevates their susceptibility to postnatal metabolic instability.

The neonatal period therefore represents a pivotal window for therapeutic intervention. Prompt identification of biochemical dysregulations, facilitated by cord-blood analysis of micronutrient and hormonal profiles, is instrumental in guiding precise nutritional and endocrine interventions. Tailored supplementation regimens, incorporating vitamin D, Ca, phosphate, and vital trace elements, alongside optimized feeding protocols, have the potential to ameliorate bone mineralization, regulate glycemic control, and foster favorable growth trajectories. Systematic metabolic surveillance during the initial postnatal weeks is imperative to mitigate transient hypoglycemia, identify nascent INS resistance, and promote compensatory growth while precluding excessive adiposity.

IUGR neonates require long-term, structured follow-up with targeted management and prevention. Therefore, we have tailored some clinical take-home messages:

- IUGR newborn start their extrauterine life facing low energy storage, some micronutrient deficits, altered insulin–C-peptide signaling, which jointly impact thermoregulation, glycemic stability, bone mineralization, and immune competence
- Cord-blood serum metabolomics should be considered at birth in IUGR neonates to stratify metabolic risk and guide further supplementation
- Early management of IUGR newborns should couple optimized nutrition with targeted supplementation of vitamins and trace elements (vitamin D, calcium, phosphate, iron, zinc, magnesium, vitamin B)
- Growth and body-composition long-term follow-up is essential, because of the increased risk of insulin resistance, dyslipidemia and hypertension.

From a translational perspective, future research endeavors should focus on several key areas:

- Establishing standardized neonatal reference ranges for micronutrients and endocrine markers tailored for IUGR populations, instead of extrapolating from healthy AGA populations
- Assessing long-term health outcomes based on early nutritional interventions in IUGR neonates
- Conducting research using multi-omics approaches—epigenomics, transcriptomics, metabolomics—in cord blood and placental tissue to map how specific micronutrient and hormonal signatures in IUGR drive persistent changes on both metabolic and neurodevelopmental outcomes.
- Develop feasible algorithms that combine clinical data with cord-blood biomarkers to tailor and individualize nutrition for infants with IUGR.

## 6. Limitations and Strengths of This Work

This narrative review is subject to several limitations that merit acknowledgment. The incorporated studies demonstrate marked heterogeneity in diagnostic criteria distinguishing IUGR from SGA, gestational age distributions, sample sizes, and biochemical reference standards, thereby hindering cross-study comparability. Predominantly, investigations rely on isolated measurements—typically from umbilical cord blood—rather than serial neonatal metabolic profiling, which constrains insights into evolving early postnatal adaptations. Additionally, confounders such as maternal anemia, diabetes, BMI, inflammation, or nutritional status were variably accounted for, potentially confounding biochemical interpretations. Evidence for select micronutrients, especially magnesium and intracellular trace elements, is sparse, as are long-term follow-up studies.

Despite these limitations, this review exhibits several key strengths. It furnishes a neonate-centric synthesis of micronutrient, endocrine, and metabolic perturbations in IUGR—a domain frequently eclipsed by maternal-centric inquiries. By integrating recent evidence across multiple biological domains, this review illuminates interconnected pathways that shape both immediate neonatal outcomes and long-term metabolic risks. The inclusion of a comparative table and illustrative diagrams enhances clinical relevance and facilitates integration into neonatal care protocols. Overall, this work synthesizes the existing literature and emphasizes the need for early biochemical monitoring and tailored nutritional interventions in IUGR neonate management.

## Figures and Tables

**Table 1:** Summary of cord-blood and early neonatal biochemical and endocrine differences between AGA, IUGR/SGA newborns, synthesizing the main micronutrient, mineral, and hormonal alterations discussed in Section 4.1, Section 4.2, Section 4.3, Section 4.4, Section 4.5 and Section 4.6. Legend: ↓ = lower in IUGR/SGA; ↑ = higher; ↔ = similar.

Domain/Micronutrient or Hormone	Marker (Cord Blood/Early Neonatal)	Pattern in IUGR/SGA vs. AGA	Typical Neonatal Sample/Timing	Main Clinical Implications in IUGR Newborns	Representative References
Vitamin D3 and phosphocalcic metabolism	25(OH)D	↓ significantly	Cord serum; day 1–3 serum	Low fetal vitamin D3 stores; impaired phosphocalcic homeostasis; increased risk of transient hypocalcemia and suboptimal postnatal growth	[2,3,4,5,8,9,10,11]
	Total/ionized Ca	↓ or low–normal	First 48–72 h	Transient neonatal hypocalcemia; neuromuscular irritability; need for Ca monitoring and supplementation in severe IUGR	[12,15,47]
	ALP	↑ when mineralization impaired	First week of life	Marker of high bone turnover; early indicator of MBDP, especially in preterm IUGR	[13,16,17,18]
Ca-phosphate–bone status	Phosphate	↓ in MBDP; paradoxical ↓ Pi + ↑ Ca	First weeks	Impaired bone mineralization; growth plate dysfunction; osteopenia and fracture risk	[13,16,17,18,19]
Fe status and oxidative metabolism	Ferritin	↓ consistently	Cord serum; day 1–3	Reduced fetal Fe stores; increased risk of Fe-deficiency anemia and impaired neurodevelopment; must interpret with inflammation markers	[20,21,22,23]
	Hemoglobin/hematocrit	variable, sometimes ↑	At birth	Polycythemia from chronic hypoxia; risk of hyperviscosity and hyperbilirubinemia	[20,22]
Zn and Mg	Zn	↓ in cord and serum	Cord blood; first 24–48 h	Impaired antioxidant defenses; immune immaturity; contribution to glycemic instability and poor growth	[26,27,28,29,30]
	Mg	↔ or mildly ↓	Cord or early serum	Subtle effects on neuromuscular excitability and vascular tone; overall less severe than Ca disturbances	[31,32]
	Intracellular Mg (platelets)	↓ in SGA	Cord blood platelets	Reduced intracellular Mg availability; altered cellular energy metabolism	[31]
Vitamin B9 and vitamin B12	Vitamin B9	↓ compared with AGA	Cord blood	Impaired DNA synthesis and cell proliferation; neurodevelopmental vulnerability	[33,34,35,36]
	Vitamin B12	↓ compared with AGA	Cord blood	Disrupted one-carbon metabolism; risk of hyperhomocysteinemia and metabolic programming	[33,34,35,36]
	Homocysteine	↑ where reported	Cord serum	Biomarker of impaired methylation capacity and oxidative stress	[34,36]
Endocrine–metabolic axis	INS	↓ in IUGR/SGA	Cord plasma	Hypoanabolic profile; reduced glycogen/fat stores; early neonatal hypoglycemia risk; abnormal catch-up growth	[1,42,43]
	C-pep	↓ in IUGR; ↑ in infants of diabetic mothers	Cord plasma	Direct β-cell secretion marker; differentiates hypoinsulinemic IUGR vs. hyperinsulinemic GDM	[1,42,43,45,46]
	Composite INS–C-pep profile	Low/low in IUGR vs. high/high in GDM	Cord blood	Distinguishes nutrient-deprivation vs. nutrient-excess intrauterine environments; predictive of later INS resistance	[1,42,43,45]

## Data Availability

The data presented in this study are available upon request from the corresponding author.
